# General analysis of factors influencing cataract surgery practice in Shanghai residents

**DOI:** 10.1186/s12886-018-0767-5

**Published:** 2018-04-18

**Authors:** Yi Xu, Jiangnan He, Senlin Lin, Bo Zhang, Jianfeng Zhu, Serge Resnikoff, Lina Lu, Haidong Zou

**Affiliations:** 1 Shanghai Eye Disease Prevention & Treatment Center / Shanghai Eye Hospital; Shanghai Key Laboratory of Ocular Fundus Diseases; Shanghai General Hospital; Shanghai Engineering Center for Visual Science and Photomedicine, 380 Kangding Road, Shanghai, 200040 China; 2 Brien Holden Vision Institute and SOVS, University of New South Wales, Sydney, NSW Australia

**Keywords:** Cataract surgery, Influencing factor, Economy, Policy, Questionnaire

## Abstract

**Background:**

It was reported that lack of knowledge, less confidence of medical services, commute difficulties, and poor economic conditions would be the main barriers for cataract surgery practice. The influencing factors could have changed in cities with high developing speed. Shanghai is one of the biggest cities in China and the world. The purpose of the study was to explore the factors influencing cataract surgery practice in Shanghai.

**Methods:**

This was a population-based, cross-sectional study. A total of 2342 cataract patients older than 50 years old with cataract-induced visual impairment or who had undergone cataract surgery were recruited from rural and urban areas of Shanghai. Participants accepted a face-to-face structured questionnaire. Data were collected on patient demographics, education, work, income, health insurance, awareness about cataracts disease, treatment and related medical resources and deration policy, transportation and degree of satisfaction with hospitals.

**Results:**

There were 417 patients who had received cataract surgery, 404 of them supplied complete information in the questionnaire. More female subjects (64.6%) than male subjects (35.4%) accepted cataract surgery among the 404 patients. Of the patients with cataract history, 36.4% of surgery patients were equal or older than 80. More people with urban medical insurance received surgery (*p* = 0.036). Patients who received surgery were more satisfied with local medical service (*p* = 0.032). In urban area, Lower income and difficulties with commutes were related to a higher rate of surgery.

**Conclusions:**

Cataract patients with the following features were more inclined to receive surgery: female, old age, better awareness. In urban areas low income and difficult commutes did not represent barriers for cataract surgery, probably because of appropriate cataract surgery promotion policies recent years in Shanghai. In rural areas, better healthcare reimbursement policies would likely lead to a higher uptake of cataract surgery. Further cohort studies with more controls could supply stronger evidence for our viewpoint.

## Background

According to the World Health Organization (WHO) report, cataracts were responsible for blindness in 10.8 million people around the world, or 33.4% of all blindness [1]. As such, it is a leading cause of avoidable blindness [2–4]. By 2020, the estimated cost required to treat the burden of avoidable blindness and visual impairment over 10 years will be US$23.1 billion [5]. In China, age-related cataracts are still the leading cause of treatable blindness [6, 7]. However, China has one of the lowest cataract surgery rates (CSR) in Asia [8–10], with clear disparities across different areas [11]. In 2014, the CSR was 1125 per 1,000,000 per year [12]. China’s CSR was much lower than that of developed countries and some developing areas such as Latin America [13]. In areas with low CSR, cataract surgeries are often postponed until the disease progresses to a worse condition, by which time there are more surgery risks and complications [14]. There is no standardized medical referral system in China; nearly all of the hospitals are walk-in, and a severe imbalance exists in the distribution of ophthalmologic medical resources. Cataract patients who want to receive surgery have to travel a long way if there are no cataract hospitals near their home. It was reported that [15] the following four problems could be primary barriers to basic cataract surgery: lack of knowledge about cataracts, low confidence in the quality of local medical services, transportation difficulties, and poor economic conditions. However, the major factors influencing cataract surgery may differ across various areas [16].

In recent years, rapid development has occurred in many areas of China, and an imbalance exists across various geographical districts and people with regard to medical resources, infrastructure, financial conditions, education level, health awareness status, and so on [17, 18]. Shanghai, which is undergoing dramatic urbanization, has one of the most rapid rates of development in China and in the world. The availability and affordability of medical services in Shanghai is relatively optimal compared to most developing districts of China [19, 20]. Over the past 15 years, the Shanghai government has been putting policies into practice to promote cataract surgeries. Shanghai’s cataract surgery rate has been rapidly increasing during the last 10 years. However, the administration of cataract surgery practice is still met with big problems in some populations. To analyse the major factors influencing cataract surgery, we enrolled 2342 Shanghai residents who had undergone cataract surgery or with cataract-induced visual impairment whose presenting visual acuity (PVA) was less than 20/40, and collected various information on population features, social economics, medical resources and patients’ health awareness status. The aim was to investigate the possible reasons for unoperated cataracts, which cause vision impairment, in a population-based study.

## Methods

### Study setting and population

This study was approved by the Ethics Committee of the Shanghai Eye Disease Prevention & Treatment Center. It was carried out in accordance with the Declaration of Helsinki. It was executed from June to November 2014 [21]. The Baoshan and Xuhui districts were randomly selected as representative rural and urban areas in the present study, respectively. Cluster sampling was used based on community unit, the sample of individuals were randomly selected. The study sampling frame for each community was constructed using geographically defined clusters based on register census data. Each cluster contained a population of approximately 1000 individuals (all ages). Basing on the percentage of population of older than 50 years old, we randomly selected 24 and 19 clusters (with equal probability) from the sampling frame of Baoshan and Xuhui districts. Finally, 11,644 residents ≥50 years old were enrolled [21, 22]. Ophthalmologic examinations were performed on each subject. Those patients who had undergone cataract surgery or who presented with cataracts and presented visual acuity (PVA) in the better eye worse than 20/40 (mild visual impairment) were chosen for the questionnaire survey. The diagnostic criteria for cataract was that patients’ lens opacity was commensurate with visual impairment and no other abnormality could account for the decrease in visual acuity. The definition of cataract-induced visual impairment was: Besides cataract, there were no other abnormality could account for the decrease in visual acuity, and PVA in the better eye worse than 20/40 [22]. Participation in the study was encouraged by the mayors of the communities. A total of 2542 patients were eligible for the study, and 2342 patients accepted the questionnaire survey, 2234 of them supplied complete information required by the questionnaire. The response rate was 92%.

### District information

The Baoshan district is in the northern part of Shanghai, and the Xuhui district is located in the southwest. The land areas of Baoshan and Xuhui cover 270.99 km^2^ and 54.76 km^2^, respectively. According to the report from the “Shanghai Statistical Yearbook 2015”, the year-end populations in 2014 were 2.024 million (Baoshan) and 1.110 million (Xuhui). According to the 2014 report from the Shanghai eye diseases prevention and treatment information management system, the district CSRs were 2951 (Baoshan) and 4548 (Xuhui), and the CSR for the entire city was 3807. The details are shown in Table 1.

**Table 1:** District information and cataract medical resources in Baoshan & Xuhui districts

	Land area (km^2^)	Population (million)	CSR in 2014	Number of hospitals for cataract treatment	Number of doctors being able to do cataract surgery
Baoshan	270.99	2.024	2951	5	16
Xuhui	54.76	1.110	4548	8	120
Total	325.75	3.134	3750	13	136

### Medical resources

There are 5 hospitals in Baoshan district in which 16 cataract surgeons perform surgery regularly. Compared to Baoshan district, Xuhui district has many more hospitals and cataract doctors (8 hospitals and 120 cataract surgeons) (Table 1).

### Patients

Door-to-door recruitment was conducted and written informed consent was acquired from each participant. Ophthalmic examination and questionnaire surveys were conducted at local community healthcare centers. All of the participants were 50 years old or older. Patients underwent ophthalmic examinations performed by professional optometrists and an ophthalmologist, including distance-presenting visual acuity (PVA) tested by a retro illuminated tumbling E LogMAR chart; intraocular pressure (IOP); fundus photography; and slit-lamp examination of eyelid, ocular globe, pupillary reflex, lens grade and fundus. Cataract was assigned when lens opacity was observed, if a previous cataract surgery was reported, or if signs of cataract or previous cataract surgery were observed in slit-lamp examination. For patients who had received cataract surgery, we recorded the surgery type, IOL (intraocular lens) position, posterior capsule status and other complications. A total of 2542 cataract patients (1312 rural, 1230 urban) with PVA in the better eye worse than 20/40 were selected for the following questionnaire survey.

### Questionnaires

There were 2342 cataract patients who accepted the interview. Face-to-face visits were conducted by four primary healthcare professionals. The four professionals were well-trained before they conducted the work, and consistency checks were performed. The following information was collected during the survey:

1. Basic information: age, gender, education, work, medical insurance, family disposable income per month.
2. Patients’ awareness: awareness about cataracts (4 questions); awareness about cataract surgery, including treatment and proper surgery access, long-term complications from untreated cataracts, whether it is painful and how long it takes to recover after the surgery (7 questions); awareness about cataract medical resources and deration policy, including whether they know which hospitals in the district perform cataract surgery, knowledge about cataract surgery cost and the reimbursement policy (6 questions). Each question in this part was scored “1”, “2”, “3” or “4” when the answer was “nothing”, “a little knowledge”, “basic knowledge” or “good knowledge”, respectively. Scores from 17 questions about awareness were summed for each patient.
3. Transportation: How long does it take to travel from your home to the nearest cataract surgery hospital? How many transfer times of the transportation would you need? The reason that we identified transfer times as a potential risk factor is that most older people in China do not know how to drive or do not drive; instead, they usually take public transportation. Transfer times could therefore limit accessibility to cataract hospitals.
4. Satisfaction with local hospitals: Are you satisfied with the facilities and medical service of the hospitals nearby?

The study abided by the tenets of the Declaration of Helsinki. It was approved by the Shanghai Eye Disease Preventive & Treatment Center Committee of Ethics.

### Statistical analyses

All statistical analyses were performed using STATISTICAL ANALYSIS SYSTEM (SAS 9.2, SAS Institute, Inc., Cary, NC. USA). Statistical significance was determined at *p* < 0.05. Absolute frequency (n) and relative frequency (%) were calculated, and Pearson chi-square (χ^2^) tests were used for the qualitative variables. Means and SDs (mean ± SD) were calculated, and logistic analysis and univariate ANOVA were applied to the quantitative variables. Multivariate logistic regression models were constructed to assess the association with surgery status, including gender, age, income, education, social insurance, hospital accessibility, satisfaction about hospital facilities, satisfaction about medical service, awareness score and district as the independent variables. The subgroup analysis within rural area or urban area about the association with surgery status were also shown. Odds ratios (ORs), 95% confidence intervals (CIs), and *p* value are also presented in Table 4.

## Results

### Clinical information derived from questionnaire interview

All of the selected patients underwent ophthalmologic examination. PVA, IOP and lens status are shown in Table 2. Most of the patients in rural and urban areas had PVA between 20/63 to < 20/40. Of patients in rural and urban areas, 13.2% and 15.4%, respectively, had PVA worse than 20/400. The average IOPs were 16.9 ± 3.9 mmHg in rural patients and 15.8 ± 4.0 mmHg in urban patients (*p* = 0.12). Among the 2342 interviewees, there were 417 patients who had received cataract surgery and 1925 patients had not.

**Table 2:** Ophthalmologic examination of subjects accepted questionnaire interview in rural and urban

	Rural *n* = 1312	Urban *n* = 1230	*P* value
PVA			0.16
< 20/40–20/63	803(61.2%)	758(61.5%)	
20/63–20/200	312(23.8%)	259(21.1%)	
20/200–20/400	24(1.8%)	24(2.0%)	
< 20/400	173(13.2%)	189(15.4%)	
IOP (mmHg)	16.9 ± 3.9	15.8 ± 4.0	0.12
Lens status			0.004
cataract	1084(82.6%)	937(83.6%)	
IOL	129(10.5%)	176(15.7%)	
aphakic	3(0.2%)	3(0.3%)	
others	5(0.4%)	5(0.4%)	

### Information about gender, age, family income, education, medical insurance type, commute, satisfaction, awareness and district of patients who had received or not received cataract surgery

Two thousand two hundred thirty-four patients supplied complete information required in the questionnaire. It showed that more female subjects (64.6%) than male subjects (35.4%) accepted cataract surgery among the 404 patients who had surgery history. Of the 404 patients, 36.4% of surgery patients were equal or older than 80. However, the most common age range in no surgery population were 60 to 70 years old (34.9%) and 70 to 80 years old (33.6%). The constituent ratio of age in surgery group and no surgery group were significantly different (*p* < 0.0001). There were no significant differences between patients who had received surgery and those who had not with regard to family income level. For patients who received surgery, 92.1% had urban medical insurance; for patients who did not receive surgery, 87.0% had urban medical insurance (*p* = 0.036). Commute times were longer among those who had received surgery (*p* = 0.0115). Patients in the surgery group were more satisfied with local medical service than people in the “no surgery” group (*p* = 0.0048). Most of the patients in the surgery group were from urban area (55.2%), whereas patients from rural area composed a larger portion (54.5%) of the “no surgery” group (*p* = 0.0004). Details are shown in Table 3.

**Table 3:** Gender, age, family income, education, medical insurance type, commute, satisfaction, awareness, district of patients accepted and unaccepted cataract surgery

	Cataract surgery history		
	Yes	No	*P* value
	*n* = 404	*n* = 1830	
Gender [n(%)]			0.0127
Male	143 (15.6%)	771 (84.4%)	
Female	261 (19.8%)	1059 (80.2%)	
Total	404 (17.8%)	1830 (82.2%)	
Age [n(%)]			< 0.0001
50- < 60	30 (14.6%)	176 (85.4%)	
60- < 70	94 (12.8%)	638 (87.2%)	
70- < 80	133 (17.8%)	614 (82.2%)	
≥ 80	147(26.8%)	402 (73.2%)	
Total	404 (17.8%)	1830 (82.2%)	
Disposable family Income per month [n(%)]			0.5624
< 1000 RMB	8 (11.6%)	61 (88.4%)	
1000–2999 RMB	207 (18.4%)	918 (81.6%)	
3000–4999 RMB	124 (18.2%)	556 (81.8%)	
≥ 5000 RMB	65 (18.1%)	295 (81.9%)	
Total	404 (17.8%)	1830 (82.2%)	
Education [n(%)]			0.0323
illiteracy	70 (16.9%)	344 (83.1%)	
Primary school	103 (19.5%)	424 (80.5%)	
middle school	179 (16.6%)	902 (83.4%)	
College and above	52 (24.5%)	160 (75.5%)	
Total	404 (17.7%)	1830 (82.3%)	
Medical Insurance [n(%)]			0.0363
No insurance	1 (5.6%)	17 (94.4%)	
Urban medical insurance	372 (18.9%)	1593 (81.1%)	
Rural medical insurance	21 (11.9%)	156 (88.1%)	
Non-local medical insurance	10 (13.5%)	64 (86.5%)	
Total	404 (17.8%)	1830 (82.2%)	
Transfer times [n(%)]			0.0115
0	181 (15.7%)	970 (84.3%)	
1	164 (20.7%)	630 (79.3%)	
≥ 2	59 (20.4%)	230 (79.6%)	
Total	404 (17.8%)	1830 (82.2%)	
Satisfaction about facilities of hospitals nearby [n(%)]			0.0319
Very satisfied	118 (21.1%)	441 (78.9%)	
Other	286 (17.1%)	1389 (82.9%)	
Total	404 (17.8%)	1830 (82.2%)	
Satisfaction about service of hospitals nearby [n(%)]			0.0048
Very satisfied	127 (22.0%)	451 (78.0%)	
Other	277 (16.7%)	1379 (83.3%)	
Total	404 (17.8%)	1830 (82.2%)	
District [n(%)]			0.0004
Rural	181 (15.4%)	998 (84.6%)	
Urban	223 (21.1%)	832 (78.9%)	
Total	404 (17.8%)	1830 (82.2%)	
^a^Awareness evaluation score			< 0.0001
Mean (standard deviation)	55.0 (23.4)	38.8 (24.2)	
	404	1830	

Pearson Chi-square (χ^2^) test and ^a^type III ANOVA test were used for qualitative and quantitative variables separately

In order to investigate different personal reasons for unacceptance of cataract surgery, we designed a question in the questionnaire: Why haven’t you accepted cataract surgery previously before the survey? There were 287 patients supplied their answers. Several kinds of answers were collected. The most frequent answers were that “I don’t think that cataract has much influence on my life. My present visual acuity still makes do” and “I did not know that my visual acuity was impaired by cataract”. Details were shown in Table 4.

**Table 4:** The reason for that patients did not accept cataract surgery treatment before the survey

	District		*P* value
	Rural (*n* = 160)	Urban (*n* = 180)	
Why haven’t you accepted cataract surgery previously before the survey?			< 0.0001
I did not know that my visual acuity was impaired by cataract.	4 (3.3%)	29 (17.5%)	
I don’t think that cataract has much influence on my life. My present visual acuity still makes do.	79 (65.3%)	74 (44.6%)	
I did not know that cataract could be treated by surgery.	1(0.8%)	5 (3.0%)	
I am worried about the effect of cataract surgery, especially the possibility of resurgence.	5 (4.1%)	21 (12.7%)	
I am worried about that I could not be able to work after surgery.	0 (0.0%)	11 (6.6%)	
I am worried about that nobody would take care of me during perioperative period.	0 (0.0%)	4 (2.4%)	
It was inconvenient for me to go to hospital and ask for help.	2 (1.7%)	3 (1.8%)	
I don’t trust on the surgery treatment.	0 (0.0%)	2 (1.2%)	
I could not afford the surgery cost.	6 (5.0%)	3 (1.8%)	
Religion reasons	0 (0.0%)	0 (0.0%)	
Others	24 (19.8%)	14 (8.4%)	
Total	121	166	

Pearson Chi-square (χ2) test

### Correlations between gender, age, family income, education, medical insurance type, commute, satisfaction, awareness, district and surgery status among cataract patients with vision impairment

To investigate the correlations between possible factors influencing cataract surgery among interviewees, we performed multivariable linear regression analysis. The results indicated that gender, age, awareness, district type, family income and commute time were significantly correlated with cataract surgery. Patients with the following features were more likely to receive cataract surgery: female (OR(95%CI) for male = 0.649 (0.506, 0.833), *p* = 0.0007), over 80 years old (OR(95%CI) = 2.521(1.535, 4.140), *p* = 0.0003), better awareness (OR(95%CI) = 1.034 (1.028, 1.040), *p* < 0.0001), lower family income (OR(95%CI) for income ≥5000 = 0.579 (0.400, 0.837), *p* = 0.0036) and longer commute time (OR(95%CI) for one transfer = 1.344 (1.033, 1.749), *p* = 0.0278). The latter two features represent a significant departure from several previous reports. To clarify the reasons that patients with lower income and inconvenient commutes were more prone to receive cataract surgery than people with higher income and more convenient transportation conditions, we further analysed patients from rural and urban areas separately. We found that these phenomena existed in urban but not rural residents. Details are shown in Table 5.

**Table 5:** Logistic regression of the correlation between gender, age, family income, education, medical insurance type, commute, satisfaction, awareness, district and surgery status

Factors		Rural & urban				Rural				Urban			
		OR	95% CI		*P* value	OR	95% CI		*P* value	OR	95% CI		*P* value
Gender	Female	reference				reference				reference			
	Male	0.649	0.506	0.833	0.0007	0.635	0.439	0.918	0.0158	0.660	0.467	0.931	0.0178
Age	50- < 60	reference				reference				reference			
	60–69	0.801	0.494	1.298	0.3675	0.627	0.340	1.155	0.1343	1.259	0.548	2.893	0.5876
	70–79	1.182	0.733	1.908	0.4931	0.875	0.469	1.632	0.6739	1.872	0.829	4.229	0.1313
	> 79	2.521	1.535	4.140	0.0003	1.841	0.947	3.582	0.0721	4.132	1.808	9.444	0.0008
Income	≤2999	reference				reference				reference			
	3000–4999	0.720	0.545	0.950	0.0203	0.731	0.477	1.120	0.1501	0.709	0.486	1.032	0.0728
	≥ 5000	0.579	0.400	0.837	0.0036	0.904	0.484	1.688	0.7506	0.501	0.313	0.804	0.0042
Education	illiteracy	reference				reference				reference			
	primary school	1.282	0.880	1.867	0.1963	1.154	0.693	1.921	0.5814	1.485	0.831	2.652	0.1816
	middle school	1.104	0.751	1.622	0.6159	0.923	0.536	1.589	0.7735	1.325	0.744	2.360	0.3391
	college and above	1.375	0.831	2.275	0.2151	0.867	0.402	1.869	0.7151	2.141	1.043	4.392	0.0379
Social insurance	without insurance &non-local insurance	0.762	0.382	1.522	0.4419	0.747	0.330	1.693	0.4855	0.718	0.188	2.744	0.6284
	urban health insurance	reference				reference				reference			
	rural health insurance	0.904	0.526	1.555	0.7158	0.812	0.446	1.479	0.4956	2.875	0.500	16.515	0.2366
Hospital accessibility	through bus	reference				reference				reference			
	one transfer	1.344	1.033	1.749	0.0278	1.143	0.762	1.716	0.5185	1.466	1.030	2.0872	0.0336
	≥ twice transfer	1.419	0.988	2.039	0.0581	1.120	0.650	1.931	0.6826	1.760	1.068	2.901	0.0266
Satisfaction about hospital facilities	others	reference				reference				reference			
	satisfied	0.667	0.369	1.203	0.1780	0.654	0.284	1.506	0.3184	0.676	0.285	1.605	0.3747
Satisfaction about medical service	others	reference				reference				reference			
	satisfied	1.632	0.918	2.903	0.0952	1.363	0.617	3.012	0.4435	1.786	0.756	4.219	0.1858
Awareness score	–	1.034	1.028	1.040	< 0.0001	1.039	1.030	1.047	< 0.0001	1.031	1.023	1.039	< 0.0001
District	Baoshan	reference				–				–			
	Xuhui	1.524	1.181	1.967	0.0012								

The Logistic analysis (full model)

## Discussion

Surgery remains the major option for cataract treatment. However, many patients in developing countries only seek cataract surgery in later stages of cataract progression. Severe complications, such as phacolytic and phacomorphic glaucoma, could occur at these later stages. In this study, patients who had visual impairment caused by cataracts completed a questionnaire survey. The survey was executed in the form of a face-to-face interview. Compared to some other studies [23–25] that were conducted by telephone, this form of survey was more appropriate for collecting broader information.

To ensure the necessity of cataract surgery in those patients, we performed ophthalmologic examination of all interviewees. Subjects suffering from other visual impairments, such as high myopia, corneal opacity, age-related macular degeneration, diabetic retinopathy and glaucoma, were excluded, as these comorbidities could confound our results. With regard to lens status, there were more “IOLs” implanted in Xuhui than in Baoshan (15.7% vs 10.5%, respectively, *p* = 0.004). This result was in accordance with the fact that the surgery rate was lower in Baoshan district.

There are many factors influencing cataract surgery in rural and urban populations. After referring to the reported studies and the local features of Shanghai city, we chose these factors described in the study to do the questionnaire survey. According to our results, 17.8% of subjects (417 out of 2342) had received cataract surgery. The surgery rate in patients with the following features was higher: female, over 80 years old, low family income, longer commute time, better health awareness, and urban residents. Patients with disposable family income greater than 3000 Chinese Yuan (about 470 US Dollars) per month were less willing to commit to surgery. Some of our results were similar to previous reports. However, a unique observation was that patients in urban areas with lower family income and longer commutes were even more prone to receive surgery. This is distinct from other studies which reported that economic and commute difficulties could pose a barrier to cataract surgery among patients with visual impairment. A possible reason for this may be that partial reimbursement of surgery costs by the Social Security Administration is profitable for hospitals providing medical service. Thus, this may be an important income source for many public hospitals. Before 2015, the reimbursement ratio of urban health insurance was much higher than that of rural health insurance (70% versus 50%) in Shanghai, which means that hospitals could benefit more from patients having urban health insurance. In recent years, cataract surgery promotion policies for patients from poor economic backgrounds have been widely promoted by the Shanghai government. Hospitals were encouraged to screen proper cataract patients with poor economy background to perform cataract surgeries, and there were shuttle buses from hospitals to pick up patients in inconvenient areas to surgery performing hospitals and send them back to home after surgeries. In such situations, more patients with low income and inconvenient commutes accepted cataract surgeries on the contrary. Due to the reimbursement profit gap between urban health insurance and rural health insurance, hospitals were much more willing to perform such screenings among populations in urban areas. The reasons listed above could cause more patients in urban area to receive cataract surgery even though their family income is low and their commute is inconvenient. This study indicates the important influence of government policy on cataract surgery promotion even though economic and commute difficulties still exist. Better policy support is needed for patients with rural health insurance. Visual impairment in different distance and cataract surgery practice was another important research topic. It was possible that patients with distant visual impairment may not have surgery because that they could still handle with daily life with near vision. It is worthy to do further study to investigate this topic.

According to the survey, the most frequent answers to why the patients did not accept cataract surgery were that “I don’t think that cataract has much influence on my life. My present visual acuity still makes do” and “I did not know that my visual acuity was impaired by cataract”. It could be explained by the reasons as follows:most of the patients were old people and work did not play an important role in their life, most of the patients did not ask for much for their quality of life, and their awareness about cataract needed improving. Health education could be effective for improving the awareness and cataract surgery practice in those patients.

During the investigation, we did general ophthalmologic evaluation for the patients. However, in the present study, we mainly used the clinical data to screen out those subjects with visual impairment caused by cataract and did questionnaire survey to the patients, so the basic clinical data related to cataract were shown, including visual acuity, intraocular pressure and lens status. In further study derived from the investigation, we will focus on the clinical examination and more clinical data would be analysed.

Patients’ health awareness is an important factor influencing surgery in general. Our results show that the surgery rate was higher among patients with better health awareness level, which in turn demonstrates the importance of health awareness in promoting cataract surgery. Since 1998, a large number of public health system building projects have been executed and promoted by the Shanghai Municipal government, the Center for Disease Control and the Shanghai Eye Disease Prevention & Treatment Center. Patient education has been widely promoted at each community health care centre. This work has improved health awareness in both rural and urban areas and has promoted cataract surgery in general.

The patients included in the study have different awareness status about the cataract disease. The awareness status was influenced by various reasons, such as patients’ education level, the health literacy, the health service access, and the health service quality [26]. All those factors could have influence on the patients’ awareness and the surgery practice, and it is very important to clarify the influence of the health service access on the awareness. We will do further study to discuss the influence of various factors on awareness on surgery practice.

## Conclusions

Shanghai is the city with the largest population in China, and it is one of the biggest cities worldwide. As a municipality, its administrative level is equal to that of a province in China. The scale and importance of this city indicates the degree to which our study may be representative of many metropolitan cities and their adjacent rural areas in China. In order to prove the effectiveness of government policies, further cohort studies with more controls will be needed, which could supply stronger evidence to support our viewpoint. This study provides information for policymakers to consider how to increase cataract surgery rates and reduce vision impairment caused by cataracts across the entire country of China.

## Abbreviations

CSR: Cataract surgery rate
PVA: Presented visual acuity
